# Intimate partner violence among African American and African Caribbean women: prevalence, risk factors, and the influence of cultural attitudes

**DOI:** 10.3402/gha.v7.24772

**Published:** 2014-09-12

**Authors:** Jamila K. Stockman, Marguerite B. Lucea, Richelle Bolyard, Desiree Bertand, Gloria B. Callwood, Phyllis W. Sharps, Doris W. Campbell, Jacquelyn C. Campbell

**Affiliations:** 1 Division of Global Public Health, Department of Medicine, University of California, San Diego La Jolla, CA, USA; 2 Department of Community-Public Health, Johns Hopkins University School of Nursing, Baltimore, MD, USA; 3 Caribbean Exploratory NIMHD Research Center, School of Nursing, University of the Virgin Islands, St. Thomas, US Virgin Islands

**Keywords:** intimate partner violence, intimate partner abuse, cultural attitudes, African American, African Caribbean, women

## Abstract

**Background:**

Women of African descent are disproportionately affected by intimate partner abuse; yet, limited data exist on whether the prevalence varies for women of African descent in the United States and those in the US territories.

**Objective:**

In this multisite study, we estimated lifetime and 2-year prevalence of physical, sexual, and psychological intimate partner abuse (IPA) among 1,545 women of African descent in the United States and US Virgin Islands (USVI). We also examined how cultural tolerance of physical and/or sexual intimate partner violence (IPV) influences abuse.

**Design:**

Between 2009 and 2011, we recruited African American and African Caribbean women aged 18–55 from health clinics in Baltimore, MD, and St. Thomas and St. Croix, USVI, into a comparative case-control study. Screened and enrolled women completed an audio computer-assisted self-interview. Screening-based prevalence of IPA and IPV were stratified by study site and associations between tolerance of IPV and abuse experiences were examined by multivariate logistic regression analysis.

**Results:**

Most of the 1,545 screened women were young, of low-income, and in a current intimate relationship. Lifetime prevalence of IPA was 45% in St. Thomas, 38% in St. Croix, and 37% in Baltimore. Lifetime prevalence of IPV was 38% in St. Thomas, 28% in St. Croix, and 30% in Baltimore. Past 2-year prevalence of IPV was 32% in St. Thomas, 22% in St. Croix, and 26% in Baltimore. Risk and protective factors for IPV varied by site. Community and personal acceptance of IPV were independently associated with lifetime IPA in Baltimore and St. Thomas.

**Conclusions:**

Variance across sites for risk and protective factors emphasizes cultural considerations in sub-populations of women of African descent when addressing IPA and IPV in given settings. Individual-based interventions should be coupled with community/societal interventions to shape attitudes about use of violence in relationships and to promote healthy relationships.

Intimate partner violence against women (IPV) continues to be a significant public health problem with adverse health consequences for women in the United States (US) of all backgrounds (1–3). However, women of African descent are disproportionately affected with 43.7% of non-Hispanic Black women (3) reporting a lifetime experience of rape, physical violence, and/or stalking by an intimate partner (1) compared to 34.6% of non-Hispanic White women, findings supported by multiple other US studies (4–9). Whether prevalence of IPV varies for women of African descent in the US (i.e. African American) and the US territories (i.e. African Caribbean) is unknown.

Limited IPV prevalence data exist for women of African descent in the US territories. Only the CDC Behavioral Risk Factor Surveillance System (BRFSS) includes the US territories, showing a lifetime IPV prevalence of 22.5% in the US Virgin Islands (USVI) and 19.5% in Puerto Rico (8). In other Caribbean countries with substantial African Caribbean populations, high rates of physical IPV (45.3–50%) and sexual IPV (52.8–72.6%) have been reported (10). A recent comparative analysis of data on IPV from population-based surveys conducted in 12 Latin American and Caribbean countries reported substantial lifetime (13.4–17.2%) and past-year (6.5–12%) prevalence of physical violence (11).

Cultural norms may influence the reporting of IPV (12) among women of African descent. Specifically, African American women are generally socialized to sacrifice individual desires for familial integration and survival within society and are perhaps more tolerant of IPV (5). For African Caribbean women, customs are rooted heavily in Caribbean culture, with male dominance and distinct gender roles clearly evident in intimate relationships, potentially contributing to more tolerance of IPV (13).

Given the heterogeneity among Black Americans, we need to consider within-group differences to develop more culturally tailored partner abuse prevention efforts (4). As such, our study assessed prevalence of physical, sexual, and psychological intimate partner abuse (IPA) among African American women in the US and African Caribbean women in the USVI. We examined the influence of IPV attitudes on abuse prevalence in both populations. Understanding IPV patterns and perceptions among these women can better inform practitioners and advocates, ensuring culturally appropriate interventions.

## Methods

### Study setting

#### Baltimore, MD, USA

Baltimore is the largest city in Maryland. In 2011, the estimated population of 619,493 was 64% Black, 32% white, and 4% some other race (14). The median household income was $23,333 USD. Seven percent of the population was foreign born (14).

#### U.S. Virgin Islands

The USVI is an unincorporated US territory, consisting of three main islands (St. Thomas, St. Croix, and St. John) and approximately 50 smaller islands. In 2012, the estimated population of 105,275 was 76% Black, African American, or of African ancestry; 13% white; and 11% some other race (15). The median household income was $41,834 USD. Approximately 70% of the population has U.S. citizenship. The immigrant rate was 4.8 per 1,000 persons (15).

### Study design

Between 2009 and 2011, we conducted a comparative case–control study to examine abuse status and associated health outcomes among African American and African Caribbean women in Baltimore, MD, and St. Croix and St. Thomas, USVI. The current study examined the: 1) screening-based prevalence of intimate partner physical, sexual, and psychological abuse among the entire sample who were screened and eligible based on race and 2) associations between IPV-tolerant attitudes and abuse experiences among the enrolled cases and controls.

### Data collection

Recruiting from primary care, prenatal, or family planning clinics, we enrolled women aged 18–55 years who had an intimate relationship within the past 2 years and who identified as being of racial or ethnic heritage that included African descent. Following informed consent, women were screened via an audio computer-assisted self-interview (ACASI). Of the 1,579 women screened, 1,545 met the inclusion criteria on age and racial background, and 1,424 reported a partner in the past 2 years. Of the 1,424 eligible women, 11 did not complete the survey. The remaining women completed a full survey via ACASI, which included questions about individual and partner sociodemographics, IPA history, and attitudes about abuse. Women completed the survey in 30 min, in private areas in the clinics. Participants received a $20 USD gift card upon completion of the interview.

### Measures

#### Abuse history

IPV was defined as a pattern of physical and/or sexual assault (and threats thereof) from a current or former intimate partner within a context of coercive control (16, 17). The broader term IPA included psychological abuse, also detrimental to women's health (18). Abuse prevalence was assessed by timing (lifetime or past 2 years) and type (physical, sexual, and psychological). Lifetime IPA and IPV were established using the Abuse Assessment (19) and Women's Experiences with Battering (WEB) (20, 21) scales. Past 2 year (recent) IPV was measured with the Severity of Violence Against Women Scales (22). Women positive for physical IPV responded positively to physical violence questions and indicated that the abuser was a current or former partner. Those who responded positively to sexual violence questions (e.g. being forced into sexual activities, having unwanted sex through threats or physical force) by a current or former partner were classified as experiencing sexual IPV. Women scoring more than 19 on the WEB were considered positive for psychological abuse. These categories were not mutually exclusive; most (67%) reported multiple types of partner abuse.

#### Sociodemographic factors

For those meeting age and race eligibility criteria, we collected sociodemographic information (e.g. age, education, birthplace).

#### Cultural attitudes on tolerance of IPV

Cases and controls shared their cultural attitudes on IPV tolerance, through measures adapted from the World Health Organization Multi-country Study on Women's Health and Domestic Violence against Women (23). Women provided their opinions of when, if ever, a man is justified in hitting his partner. Their responses were dichotomized into personally tolerant or not tolerant of violence. Women also reported how acceptable IPV was in their community; ‘never okay’ was considered an IPV-intolerant community, while ‘occasionally’ to ‘always okay’ were considered IPV-tolerant communities.

### Statistical analysis

For the entire racially and age eligible population (n=1,545), univariate statistics were used to report prevalence of lifetime IPA, lifetime IPV, and recent IPV, and to describe sociodemographic characteristics by lifetime and recent IPV status. Wilcoxon's Rank Sum tests and *t*-tests were used for continuous normally and non-normally distributed variables. Pearson's Chi-square was used for binary variables. We performed univariate and multivariate logistic regressions to identify sociodemographics associated with lifetime and recent IPV. All variables attaining significance levels <10% in univariate models were included in multivariate models. We assessed model fit using the Akaike Information Criterion (AIC). Among enrolled women, we constructed additional logistic regression models, assessing the association between tolerance of IPV and abuse status.

### Ethics

The Institutional Review Boards (IRB) at Johns Hopkins Hospital and the University of the Virgin Islands granted approval for this project. The National Institutes of Health provided a certificate of confidentiality. Written informed consent was obtained from all participants.

## Results

### Sample characteristics

Of the 1,545 screened and race-eligible women, 486 (32%) were from Baltimore, 545 (35%) were from St. Thomas, USVI, and 514 (33%) were from St. Croix, USVI. The majority of the women (1,413, 91%) identified as being Black African American or African Caribbean, while 132 (9%) considered themselves mixed race with African descent. Only 241 (17%) identified as being Spanish or Hispanic African American. The average age was 29.7 years (SD 9.49). About one-fourth of the screened women (26%) were pregnant, 70% had at least one child aged ≤18 years living at home, and 76% were in a current intimate relationship. Approximately 80% had at least a high school diploma or equivalent, nearly half (48%) were employed, and 65% had health insurance including Medicaid or government subsidized insurance programs (Table 1). The majority (93%) reported an individual annual income of less than $24,000 USD.

The characteristics of the enrolled sample (n=901) were similar to that of the overall population with some differences by site. Fewer women were pregnant in St. Croix, compared to St. Thomas and Baltimore (21, 31, and 30% respectively, *p*<0.05). More women were employed in St. Thomas than both St. Croix and Baltimore (60, 44, and 42% respectively, *p*<0.01). More women in Baltimore had health insurance than women in St. Thomas and St. Croix (90, 57, and 54% respectively, *p*<0.01).

Certain sociodemographics differed between abused and non-abused women. More non-abused women had insurance (75 vs. 65%, *p*<0.01) and were in current partnerships than abused women (86 vs. 78%, *p*<0.01). More non-abused women were pregnant than abused women (33 vs. 24%, *p*<0.05), but more abused women had children (76 vs. 66%, *p*<0.01).

### Prevalence

#### Overall prevalence

Among the 1,545 screened and race-eligible women, prevalence of lifetime IPA and IPV was 40 and 27%, respectively. By site, lifetime prevalence of IPA and IPV were, respectively, 45 and 38% in St. Thomas, 38 and 28% in St. Croix, and 37 and 30% in Baltimore (*p*<0.05).

Among enrolled women reporting having an intimate partner in the past 2 years (n=1,424), overall prevalence of recent IPV and forced/coerced sex was 27 and 8%, respectively. Among these women, the prevalence of recent IPV was 32% in St. Thomas, 22% in St. Croix, and 26% in Baltimore (*p*<0.05). Prevalence of recent forced/coerced sex did not differ significantly by site (10% in St. Thomas, 8% in St. Croix, and 7% in Baltimore).

#### Type of IPA

Among women experiencing lifetime IPA (n=543), the majority reported physical and psychological abuse (31%) and all three types of abuse (30%). Women in St. Thomas (n=207) reported more combined physical and psychological abuse (44%) than those in St. Croix (24% of 177 women) and Baltimore (23% of 159 women) (*p*<0.05). Women in all three sites reported comparable prevalence of all three types of violence (29% in St. Thomas, 30% in St. Croix, and 31% in Baltimore). Significantly more women in Baltimore reported physical violence only (22%) than those in St. Thomas (7%, *p*<0.05), but not St. Croix (12%). Prevalence of sexual abuse combined with either psychological or physical abuse ranged from 6% in St. Thomas to 12% in St. Croix (9% in Baltimore) while more women in St. Croix (22%) reported psychological abuse only than in the other sites (13% St. Thomas; 15% Baltimore). Yet, these differences were not statistically significant.

#### Timing of IPA and IPV

Timing of abuse differed by site in terms of recent IPV and lifetime psychological abuse only, but not for those women exclusively experiencing distant (more than 2 years ago) IPV. Very few abused women (9% overall) reported distant IPV without recent experiences, with no site differences. At the same time, 67% of abused women overall reported recent experiences of IPV. Of the abused women in Baltimore and St. Thomas, 71 and 73% reported recent IPV, respectively, but only 57% of abused women in St. Croix reported recent IPV (*p*<0.01). For the 21% of abused women experiencing psychological abuse only (not specific to time), women in St. Croix reported higher prevalence (28%, *p*<0.01) than women in Baltimore or St. Thomas (19 and 15%, respectively).

### Sociodemographics and IPV

Having children aged ≤18 years was the only factor associated with lifetime IPV in all three sites (Table 1). In St. Thomas, women born in the US/USVI and women with a current partner born in the US/USVI were significantly more likely to have ever experienced IPV (ORs=1.85 and 1.78, respectively) than those born outside the USVI themselves and with partners born outside the USVI, respectively. In St. Croix, having a current partner was protective against ever experiencing IPV (OR=0.43; 95% CI=0.28, 0.65).

**Table 1:** Lifetime IPV and associated sociodemographics among women of African descent in Baltimore, MD, and the USVI, 2009–2011

Sociodemographics	Overall (n=1,545)	Baltimore (n=486)		St. Thomas (n=545)		St. Croix (n=514)	

	n (%)	n (%)	OR (95% CI)	n (%)	OR (95% CI)	n (%)	OR (95% CI)
Mean age (SD)	29.7 (9.5)	29.3 (9.0)	0.99 (0.97–1.01)	28.6 (8.8)	0.99 (0.98–1.02)	29.5 (8.9)	0.99 (0.97–1.01)
Education							
No HS diploma	321 (20.9%)	40 (27.2%)	Ref.	33 (16.0%)	Ref.	25 (17.5%)	Ref.
HS graduate	625 (40.7%)	62 (42.2%)	0.97 (0.60–1.56)	68 (33.0%)	0.87 (0.52–1.46)	60 (42.0%)	1.22 (0.72–2.08)
Some college	348 (22.7%)	32 (21.8%)	1.40 (0.79–2.49)	68 (33.0%)	1.20 (0.71–2.03)	34 (23.8%)	1.63 (0.89–2.98)
College graduate	240 (15.7%)	31 (8.8%)	0.69 (0.33–1.41)	37 (18.0%)	1.13 (0.62–2.05)	24 (16.8%)	1.13 (0.60–2.15)
Currently employed							
No	810 (52.5%)	94 (64.0%)	Ref.	84 (40.6%)	Ref.	78 (54.2%)	Ref.
Yes	733 (47.5%)	53 (36.1%)	0.12 (0.48–1.07)	123 (59.4%)	1.21 (0.85–1.72)	66 (45.8%)	1.14 (0.78–1.68)
Medical insurance							
No	546 (35.5%)	15 (10.3%)	Ref.	96 (46.4%)	Ref.	64 (44.4%)	Ref.
Yes	993 (64.5%)	131 (89.7%)	0.79 (0.41–1.53)	111 (53.6%)	0.97 (0.68–1.37)	80 (55.6%)	1.33 (0.91–1.97)
Current partner							
No	373 (24.1%)	41 (27.9%)	Ref.	52 (25.1%)	Ref.	51 (35.4%)	Ref.
Yes	1,172 (75.9%)	106 (72.1%)	0.71 (0.46–1.11)	155 (74.9%)	1.02 (0.68–1.52)	93 (64.6%)	**0.43 (0.28–0.65)**
Children <18 years							
No	465 (30.1%)	32 (21.8%)	Ref.	48 (23.2%)	Ref.	32 (22.2%)	Ref.
Yes	1,079 (69.9%)	115 (78.2%)	**2.10 (1.34–3.29)**	159 (76.8%)	**1.65 (1.11–2.45)**	112 (77.8%)	**1.60 (1.02–2.51)**
Currently pregnant							
No	1,146 (74.2%)	106 (72.1%)	Ref.	150 (72.5%)	Ref.	117 (81.3%)	Ref.
Yes	398 (25.8%)	41 (27.9%)	0.82 (0.53–1.25)	57 (27.5%)	1.22 (0.82–1.81)	27 (18.8%)	0.79 (0.48–1.28)
Born in US/USVI							
No	293 (19.1%)	1 (0.7%)	Ref.	49 (23.8%)	Ref.	31 (21.5%)	Ref.
Yes	1,244 (80.9%)	146 (99.3%)	3.53 (0.44–28.44)	157 (76.2%)	**1.85 (1.25–2.74)**	113 (78.5%)	1.05 (0.66–1.67)
Current partner born in US/USVI							
No	260 (22.2%)	1 (0.9%)	Ref.	37 (23.9%)	Ref.	24 (25.8%)	Ref.
Yes	910 (77.8%)	105 (99.1%)	4.96 (0.64–38.64)	118 (76.1%)	**1.78 (1.14–2.80)**	69 (74.2%)	1.36 (0.81–2.30)

OR=odds ratio; CI=confidence interval; SD=standard deviation; Ref.=reference group; USVI=US Virgin Islands. OR (CI) values that are significant at the p<0.05 level are shown in bold.

For recent IPV, older age was protective in Baltimore and St. Croix in bivariate analyses (Table 2). In Baltimore alone, women who had children aged ≤18 years were more likely to have experienced recent IPV (OR=2.38; 95% CI=1.43, 3.98). In St. Thomas alone, women who were born in the US/USVI and those who had a current partner who was born in the US/USVI were more likely to have experienced recent IPV (OR=1.76; 95% CI=1.12, 2.76 and OR=1.88; 95% CI=1.16, 3.07, respectively). In St. Croix alone, having a current partnership was protective against recent IPV (OR=0.43; 95% CI=0.26–0.72).

**Table 2:** Past two year IPV and associated sociodemographics among women of African descent in Baltimore, MD, and the USVI, 2009–2011

Sociodemographics	Overall	Baltimore		St. Thomas		St. Croix	

	n (%)	n (%)	OR (95% CI)	n (%)	OR (95% CI)	n (%)	OR (95% CI)
Mean age (SD)	29.3 (9.1)	28·1 (8.3)	**0.98 (0·96–1.00)**	27.5 (8.0)	0.98 (0.96–1.01)	27.8 (8.2)	**0.97 (0.94–0.99)**
Education							
No HS diploma	292 (20.7%)	33 (27.7%)	Ref.	26 (16.6%)	Ref.	20 (19.0%)	Ref.
HS graduate	578 (40.9%)	51 (42.9%)	0.95 (0.57–1.58)	52 (33.1%)	0.87 (0.49–1.53)	42 (40.0%)	0.96 (0.53–1.74)
Some college	322 (22.8%)	27 (22.7%)	1.42 (0.77–2.62)	52 (33.1%)	1.18 (0.66–2.11)	25 (23.8%)	1.24 (0.64–2.41)
College graduate	222 (15.7%)	8 (6.7%)	0.48 (0.20–1.11)	27 (17.2%)	1.06 (0.55–2.04)	18 (17.1%)	0.95 (0.47–1.92)
Currently employed							
No	742 (52.2%)	75 (63.0%)	Ref.	60 (38.2%)	Ref.	60 (56.6%)	Ref.
Yes	680 (47.8%)	44 (37.0%)	0.75 (0.49–1.15)	97 (61.8%)	1·23 (0.83–1.81)	46 (43.4%)	1.05 (0.68–1.62)
Medical insurance							
No	494 (34.8%)	14 (11.9%)	Ref.	75 (47.8%)	Ref.	47 (44.3%)	Ref.
Yes	925 (65.2%)	104 (88.1%)	0.62 (0.31–1.22)	82 (52.2%)	0.85 (0.58–1.24)	59 (55.7%)	1.32 (0.85–2.04)
Current partner							
No	252 (17.7%)	30 (25.2%)	Ref.	32 (20.4%)	Ref.	30 (28.3%)	Ref.
Yes	1,172 (82.3%)	89 (74.8%)	0.62 (0.38–1.02)	125 (79.6%)	0.65 (0.40–1.07)	76 (71.7%)	**0.43 (0.26–0.72)**
Children <18 years							
No	408 (28.7%)	22 (18.5%)	Ref.	36 (22.9%)	Ref.	26 (24.5%)	Ref.
Yes	1,016 (71.4%)	97 (71.5%)	**2.38 (1.43–3.98)**	121 (77.1%)	1.27 (0.81–1.97)	80 (75.5%)	1.37 (0.83–2.24)
Currently pregnant							
No	1,033 (72.6%)	83 (69.8%)	Ref.	113 (72.0%)	Ref.	82 (77.4%)	Ref.
Yes	390 (27.4%)	36 (30.3%)	0.90 (0.57–1.42)	44 (28.0%)	1·05 (0.68–1.60)	24 (22.6%)	0.97 (0.58–1.62)
Born in US/USVI							
No	244 (17.2%)	1 (0.8%)	Ref.	33 (21.0%)	Ref.	23 (21.7%)	Ref.
Yes	1,172 (82.8%)	118 (99.2%)	2.47 (0.30–20.25)	124 (79.0%)	**1.76 (1.12–2.76)**	83 (78.3%)	0.95 (0.56–1.61)
Current partner born in US/USVI							
No	260 (22.2%)	0 (0%)	–	28 (22.4%)	Ref.	19 (25.0%)	Ref.
Yes	910 (77.8%)	89 (100%)	–	97 (77.6%)	**1.88 (1.16–3.07)**	57 (75.0%)	1.41 (0.80–2.49)

OR=odds ratio; CI=confidence interval; SD=standard deviation; Ref.=reference group; USVI=US Virgin Islands; – denotes small sample size resulting in the absence of a univariate analysis. OR (CI) values that are significant at the p<0.05 level are shown in bold.

Controlling for other factors, having children aged ≤18 years remained a significant risk factor for lifetime IPV across all sites (Table 3). In St. Thomas, being born in the US/USVI remained significantly associated with lifetime IPV (AdjOR=2.02; 95% CI=1.32, 3.09). In St. Croix, having a current partner remained protective against lifetime IPV (AdjOR=0.34; 95% CI=0.22, 0.54), and older age emerged as an additional protective factor against lifetime IPV (AdjOR=0.97; 95% CI=0.94, 0.99).

**Table 3:** Factors independently associated with lifetime and recent IPV among women of African descent in Baltimore, MD, and the USVI, 2009–2011

	Lifetime IPV			Past two year IPV		

	Baltimore AdjOR (95% CI)	St. Thomas AdjOR (95% CI)	St. Croix AdjOR (95% CI)	Baltimore AdjOR (95% CI)	St. Thomas AdjOR (95% CI)	St. Croix AdjOR (95% CI)
Age	0.99 (0.97–1.02)	1.00 (0.97–1.02)	**0.97 (0.94–0.99)**	0.98 (0.95–1.01)	0.98 (0.95–1.01)	**0.95 (0.92–0.98)**
Education						
No HS diploma	Ref.	Ref.	Ref.	Ref.	Ref.	Ref.
HS graduate	1.04 (0.63–1.70)	0.70 (0.40–1.22)	1.45 (0.83–2.56)	1.01 (0.59–1.73)	0.65 (0.35–1.21)	1.07 (0.57–2.00)
Some college	1.58 (0.86–2.90)	0.95 (0.53–1.69)	1.81 (0.95–3.45)	1.59 (0.83–3.04)	0.89 (0.46–1.69)	1.24 (0.61–2.53)
College graduate	0.87 (0.41–1.84)	0.96 (0.50–1.82)	1.31 (0.66–2.61)	0.61 (0.25–1.47)	0.95 (0.47–1.95)	1.09 (0.51–2.35)
Currently employed						
No	Ref.	Ref.	Ref.	Ref.	Ref.	Ref.
Yes	0.68 (0.45–1.05)	1.31 (0.89–1.93)	1.28 (0.84–1.96)	0.77 (0.49–1.22)	1.47 (0.95–2.26)	1.30 (0.80–2.10)
Medical insurance						
No	Ref.	Ref.	Ref.	Ref.	Ref.	Ref.
Yes	0.73 (0.37–1.46)	0.77 (0.53–1.12)	1.41 (0.93–2.14)	0.58 (0.28–1.20)	0.71 (0.47–1.08)	1.39 (0.87–2.22)
Current partner						
No	Ref.	Ref.	Ref.	Ref.	Ref.	Ref.
Yes	0.64 (0.40–1.03)	0.78 (0.50–1.20)	**0.34 (0.22–0.54)**	**0.55 (0.32–0.94)**	0.61 (0.36–1.02)	**0.35 (0.20–0.61)**
Children <18 years						
No	Ref.	Ref.	Ref.	Ref.	Ref.	Ref.
Yes	**2.14 (1.34–3.43)**	**1.65 (1.08–2.52)**	**1.94 (1.19–3.16)**	**2.45 (1.43–4.18)**	1.32 (0.82–2.12)	**1.72 (1.00–2.96)**
Currently pregnant						
No	Ref.	Ref.	Ref.	Ref.	Ref.	Ref.
Yes	0.79 (0.49–1.26)	1.28 (0.84–1.96)	0.79 (0.46–1.35)	0.84 (0.51–1.39)	1.04 (0.67–1.64)	0.84 (0.48–1.47)
Born in US/USVI						
No	Ref.	Ref.	Ref.	Ref.	Ref.	Ref.
Yes	3.55 (0.43–29.28)	**2.02 (1.32–3.09)**	0.89 (0.54–1.47)	2.30 (0.27–19.57)	**1.96 (1.21–3.20)**	0.74 (0.42–1.30)

IPV=intimate partner violence; AdjOR=adjusted odds ratio; CI=confidence intervals; ref.=referent group; HS=high school; USVI=US Virgin Islands. AdjOR (CI) values that are significant at the p<0.05 level are shown in bold.

For recent IPV, differences also emerged. In Baltimore and St. Croix, women with a current partner were significantly less likely to experience recent IPV but those with children were more likely to have such an experience (see Table 3). In addition, in St. Croix, older aged women were less likely to experience recent IPV, and in St. Thomas, women born in the US/USVI were more likely to experience recent IPV compared to their counterparts (Table 3).

### Impact of cultural attitudes regarding tolerance of IPV

Community tolerance of IPV was highly correlated with IPA in initial analyses for all sites. In Baltimore, 10% of women felt their communities were accepting of IPA: 18% among abused women and 4% among those never abused (*p*>0.01). St. Thomas had the highest percentage (24%) of women perceiving their communities to be accepting of violence (30% of abused and 10% of never abused women; *p*>0.01). In St. Croix, 17% felt their communities were accepting of violence (20% of abused and 10% of never abused women; *p*<0.05). Community acceptance of IPV was independently associated with more than four- and three-fold higher odds of lifetime IPA in Baltimore and St. Thomas, respectively (see Table 4). The elevated risk for abuse for women in St. Croix did not remain significant.

**Table 4:** Multivariate logistic regression model for the association between personal and community acceptance of IPV and lifetime experiences of IPA among women of African descent in Baltimore, MD, and the USVI, 2009–2011

	Lifetime IPA			

	Overall (n=884; Pseudo R^2^=0.08) AdjOR (95% CI)	Baltimore (n=342; Pseudo R^2^=0.07) AdjOR (95% CI)	St. Thomas (n=285; Pseudo R^2^=0.13) AdjOR (95% CI)	St. Croix (n=254; Pseudo R^2^=0.08) AdjOR (95% CI)
Age group				
18–24	Ref.	Ref.	Ref.	Ref.
25–34	1.21 (0.87–1.70)	**1.84 (1.08–3.17)**	0.82 (0.44–1.55)	0.86 (0.45–1.65)
35–44	1.42 (0.93–2.18)	1.61 (0.81–3.19)	1.30 (0.52–3.21)	1.19 (0.54–2.65)
45+	1.12 (0.66–1.90)	1.57 (0.72–3.38)	0.90 (0.33–2.46)	1.40 (0.38–5.07)
Education level				
No HS diploma	Ref.	Ref.	Ref.	Ref.
HS diploma	1.24 (0.84–1.82)	1.32 (0.76–2.30)	1.19 (0.51–2.76)	0.77 (0.33–1.81)
Some college	**1.96 (1.25–3.05)**	**2.17 (1.03–4.55)**	1.40 (0.58–3.40)	1.00 (0.39–2.60)
College graduate	1.34 (0.82–2.21)	0.84 (0.38–1.87)	2.14 (0.73–6.31)	0.89 (0.33–2.40)
Current partner				
No	Ref.	Ref.	Ref.	Ref.
Yes	**0.55 (0.38–0.80)**	0.83 (0.48–1.46)	0.50 (0.22–1.11)	**0.18 (0.07–0.47)**
Perceived community acceptance of IPV				
Not accepting	Ref.	Ref.	Ref.	Ref.
Accepting	**3.42 (2.10–5.56)**	**4.34 (1.85–10.24)**	**2.89 (1.26–6.63)**	2.38 (0.96–5.94)
Personal acceptance of IPV				
Not accepting	Ref.	Ref.	Ref.	Ref.
Accepting	**4.19 (2.34–8.10)**	**3.06 (1.15–7.48)**	**12.77 (3.00–54.47)**	2.00 (0.73–5.39)

IPA=intimate partner abuse; AdjOR=adjusted odds ratio; CI=confidence intervals; Ref.=referent group; HS=high school; IPV=intimate partner violence; USVI=US Virgin Islands. AdjOR (CI) values that are significant at the p<0.05 level are shown in bold.

Personal acceptance of IPV followed a similar pattern in all sites. Overall, few women felt abuse was acceptable. In Baltimore, 7.5% of women reported IPV acceptable in certain situations in a relationship (11% abused vs. 4% never abused; *p*<0.01). In St. Thomas, 21% reported personally accepting the use of IPV in relationships (28% abused vs. 2% never abused; *p*<0.01). Of the women in St. Croix, 12% reported a personal acceptance of IPV in relationships, but the difference between abused (14%) and non-abused (7%) groups was not significant. In multivariate analyses, women who were accepting of violence were three times more likely in Baltimore (AdjOR=3.06; 95% CI=1.15, 7.48) and 12 times more likely in St. Thomas (AdjOR=12.77; 95% CI=3.00, 54.47) to report experiencing lifetime IPA.

## Discussion

Our prevalence data show a higher or comparable prevalence of recent IPV (27%) and lifetime IPA (40%) among African American women, when compared to other population-based US studies. The most recent population-based study from CDC (1) reported a 40.9% weighted prevalence of lifetime intimate partner physical violence for African American (Black, non-Hispanic) women and 12.2% for intimate partner rape. The BRFSS (8) reported 29.2% lifetime prevalence of IPV for African American women overall and 22.5% in the USVI. Some attribute the lower prevalence in the BRFSS data for all racial/ethnic groups to the limited number of questions on violence embedded in the larger BRFSS survey, and that no strategies assured privacy for responses, unlike both our study and the CDC study. Other population-based surveys (i.e. Demographic and Health Survey and the Reproductive Health Survey) now include modules on prevalence and consequences of violence against women in the Latin American and Caribbean region (11).

Our study's higher prevalence might be related to the setting and survey administration. Prevalence of IPV among women in health care settings would be expected to be higher than population-based data since abused women often have more health problems and therefore might be more highly represented in clinic settings (24). Moreover, our study used an ACASI system for self-reporting of abuse, which can increase disclosure of traumatic experiences (25).

Fear and stigma have been found to be associated with IPV disclosure (12, 26). St Croix has a strong Cruzan cultural and family orientation. The desire to protect the family from ridicule may prevent disclosure of IPV among St. Croix women, despite more support resources being available in St. Croix than in St. Thomas. This may explain part of the distinct profiles in reporting on the islands with St. Croix having high rates of psychological abuse/controlling behavior and St. Thomas having the highest rates of IPV.

Few women demonstrated long-term separation from violence. The majority remain in or return to abusive relationships, increasing the possibility of severe and potentially lethal violence. Targeted interventions should help women break the violent cycle in a current or sequential relationship. More women in St. Croix had broken the cycle, perhaps reflecting the lower community tolerance of abuse there. The increased availability of domestic violence resources on St. Croix compared to St. Thomas might provide women more options to stop the violence.

Consistent with prior research, having children, being younger, and having a locally born partner were correlates of recent IPV. Some sociodemographics (e.g. US/USVI born partners) are difficult to address and indicate the need for increased efforts to encourage social norms change. Others (e.g. children) could benefit from a multi-pronged approach to protect women from repeated violence and to prevent multi-generational violence. Specifically, younger age was associated with recent IPV, which emphasizes the importance of screening and early interventions with young people regarding healthy relationships.

The vast majority of women in all settings felt that IPV was never or rarely acceptable and that their communities were not tolerant of IPV. However, independent of other factors, individual and community IPV acceptance were main drivers in elevated risk for IPA for these women, an association supported by other Caribbean (11) and global data (23). Perhaps abused women view their communities as more tolerant as a way to rationalize the abuse, or perhaps their perception of community tolerance is realistic.

The difference between St. Croix and St. Thomas in perceived community tolerance is striking and may reflect community differences concerning domestic violence. The prevalence of IPV is often significantly higher among urban compared with rural women (11), and St. Thomas is more urban than St. Croix. In addition, abused women in IPV-tolerant communities exhibit less help-seeking behavior. Previously, we found women in St. Croix were more likely to believe that help was available from a variety of community resources than women in St. Thomas (27). Perceived stigma, fear, and potential relationships with responders may act as barriers to help-seeking by women in St. Thomas, which would align with reasons for not seeking help, including shame, fear of retaliation, not knowing where to go, and not believing anyone would help (11).

Measures of violence and abuse should include adequate assessments of both lifetime and recent abuse to promote comparison of prevalence data across countries and territories with high representation of women of African descent. Although our study is the first attempt to document abuse prevalence accounting for the heterogeneity of the Black race in two distinct settings, our findings are not generalizable to Black women in other settings (e.g. African born Blacks). More research is needed to document the comprehensive abuse epidemiology among these vulnerable women.

Our findings demonstrate that individual-based interventions should be coupled with community/societal interventions to shape attitudes about use of violence in relationships and to promote healthy relationships. Coalitions of community, governmental, and societal institutions can work collaboratively to develop and evaluate comprehensive approaches to preventing and improving responses to violence. Efforts to reform and transform policies and practices of major institutions are needed to strengthen their response to violence against women, including policies and practices of the health care, judiciary, schools and social services agencies.
